# Supplementary material for: SV40 Transfected Human Anterior Cruciate Ligament Derived Ligamentocytes—Suitable as a Human in Vitro Model for Ligament Reconstruction?
Source: Int J Mol Sci. 2020 Jan 16;21(2):593. doi: 10.3390/ijms21020593 (PMC7014138; doi:10.3390/ijms21020593)

Supplemental Figure 1: Extracellular matrix of the donor tissue and Mohawk immunoreactivity of isolated non-transfected and SV40 transfected cells. A: Resorcin-Fuchsin staining of the ACL tissue to visualize elastic fibers. B: fibronectin immunolabeling of the ACL tissue. C, D: Mohawk expression in non-transfected (C) and transfected cells (D). A: Scale bar: 100 µm, B-D: Scale bars: 50 µm.

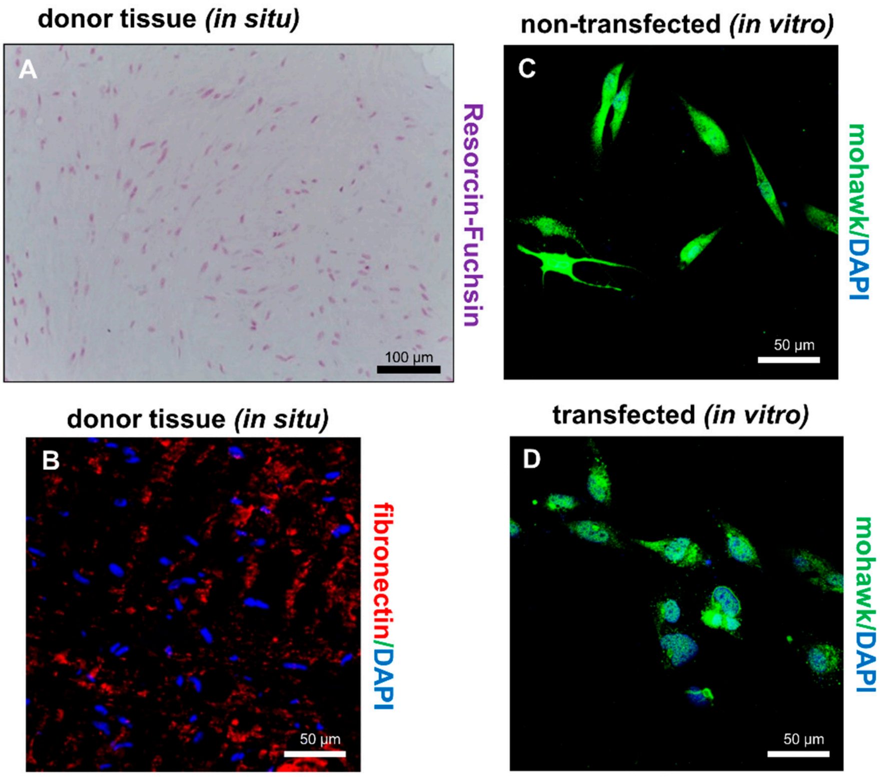

Supplement: Supplementary file 1 [file ijms-21-00593-s001.pdf]
